# Effect of race on Gaze Cueing in adults with high and low autistic traits

**DOI:** 10.1186/s40359-023-01307-y

**Published:** 2023-09-15

**Authors:** Paola Ricciardelli, Noemi Pintori

**Affiliations:** 1 Department of Psychology, University of Milan – Bicocca, Piazza Dell’Ateneo Nuovo, 1, 20126 Milan, Italy; 2 Milan Centre for Neuroscience, Milan, Italy; 3 Department of Clinical and Experimental Sciences, University of Brescia, Viale Europa, 11, 25123 Brescia, Italy

**Keywords:** Gaze-cueing effect, Ingroup bias, Implicit race bias, High and Low autistic traits

## Abstract

**Background:**

Observing the direction of gaze of another person leads to shifting of attention in the same direction (gaze-cueing effect – GCE), a social-cognitive ability known as joint or social attention. Racial attitudes can influence the magnitude of GCE since it has been shown that White people showing a strong race ingroup preference follow the gaze only of White, and not Black, faces. Individuals with high autistic traits have difficulties in social-cognitive abilities that can disrupt the learning of socially shared racial attitudes. Our aim was to investigate in White Italian adults whether individuals with higher autistic traits (measured by the Autism Spectrum Quotient) show reduced implicit racial bias (measured by the Implicit Association Test) and if this bias would lead to differences in the gaze cueing effect (GCE) triggered by gaze direction of faces of different races (measured by the Gaze Cueing Task).

**Methods:**

In an online study, participants (*N* = 165; 132 females; Mean age = 22.9; SD = 4.76) filled in the Autism Spectrum Quotient (AQ) questionnaire, then performed a Gaze Cueing Task, followed and by an Implicit Association Test.

**Results:**

Linear regression and linear mixed model analyses showed in the IAT task the presence of the same implicit ingroup bias for all participants, which was not predicted by the AQ score, while in the Gaze Cueing Task the GCE differed depending on the AQ score of the participants. Specifically, participants with low-medium, medium, and medium–high autistic traits (AQ = -1SD; AQ = mean; AQ =  + 1SD respectively) presented the GCE for both ingroup and outgroup cueing faces, whereas participants with high autistic traits (AQ =  + 2SD) only for ingroup faces.

**Conclusions:**

In White Italian adults the presence of an implicit ingroup bias seems to influence the GCE, but it is not always true that the individuals showing an implicit ingroup bias do not orient their attention in the direction of gaze of the outgroup individuals. Instead, the GCE seems to be modulated by the level of autistic traits. That is, individuals with higher autistic traits seem to prioritize joint attention with only their ingroup members.

## Background

Race, gender, and age are some of the social categories through which individuals categorize others. Person perception and social categorization play an important role in joint attention and interpersonal interactions (e.g., [1, 2]). Joint (or social) attention is the orientation of our attention toward the direction of gaze of others [3, 4]. In adults, it results in being quicker to discriminate a stimulus when it appears at the gazed-at spatial location (gaze-cueing effect—GCE). It develops early in life [5] and it is essential for developing referential communication and understanding the intentions of others since eyes serve as a social signal to infer the internal mental states of others (e.g., emotions) and predict their future behaviours.

Recently, relevant social factors such as, for example, dominance (i.e., dominant individuals exert a greater GCE) [6], age (i.e., faces of similar age of the observer elicit a greater GCE) [7], familiarity with the cueing face (i.e., more familiar faces elicit an enhanced GCE) [8], and the gender of the observers (i.e., females show a greater GCE than males; and females with low or averaged level of competition show an enhanced GCE for competitive contenders than for cooperative) [9, 10], have been reported to modulate the GCE. In other words, a key role in gaze cueing is played by who the person we observe is (e.g., [1, 7]).

Race, as well as other social categories, is associated with stereotypes and attitudes (e.g., [11, 12]). Evaluating people more or less positively on the basis of their race could influence how much we are susceptible to their gaze direction. Intuitively, in fact, it is plausible that, if we like someone less and if we have a negative attitude toward somebody, it is less likely we pay attention to them and orient our attention in the same direction of their gaze. It is known that in contexts in which in the social hierarchy White people have a higher position than Black people (e.g., in USA and in Italy), White people have more positive implicit attitudes toward their own ingroup members compared to Black people (e.g., [13, 14]). In a study investigating empathic sensorimotor resonance for pain, Avenanti, Sirigu and Aglioti [13] showed that White Italian participants had a greater ingroup bias (i.e., having a more positive implicit attitude toward their own ingroup members) than Black African participants, and that participants with higher implicit ingroup preference exhibited greater differences in the empathic response to ingroup and outgroup members. Ingroup preference, thus, seems to affect a very basic social process like the empathic response [13] and might be a good candidate as a factor capable of affecting another social process such as joint attention.

Interestingly, in a joint attention study in which White and Black participants were requested to discriminate a peripheral letter appearing either to the left or to the right of a task-irrelevant White or Black face, Pavan et al. [15] found that Black participants shifted attention in the same direction of the face averted gaze (i.e., gaze cueing effect) both for ingroup (i.e., Black) and outgroup (i.e., White) faces, whereas White participants shifted attention toward gaze direction only in response to faces of their own ingroup (i.e., White faces). Therefore, it is plausible that differences between Black and White people in the implicit ingroup preference (i.e., White people showing a greater ingroup preference) can also be reflected in the orienting of attention (e.g., [13, 14]). Specifically, the implicit attitudes toward ingroup and outgroup could influence the gaze cueing effect when faces of different races are used as cueing faces. Pavan et al. [15] did not include in their experimental design a direct measure of the implicit racial attitudes and explained the different performance of White and Black participants more in terms of differences in the relative social status (i.e., in the Italian context, Black people are perceived as a low status group than White people). In order to investigate the role of racial attitudes in joint attention, we decided to include in our study a measure of implicit racial attitudes to shed light on their possible influence on the gaze cueing effect (GCE).

People with Autism Spectrum Condition (ASC) [16] behave differently to most people; they show limited interest in social stimuli (e.g., [17]) and, depending on the degree of this condition, they find it hard to deal with social situations since they have reduced social skills (including language development, gaze perception and joint attention). Relevant to the present study, they show atypical social learning, which is an important mechanism in the formation of stereotypes and attitudes (e.g., [18, 19]). Since stereotypes and attitudes are socially transmitted, it is possible that people with ASC lack stereotypes and attitudes acquisition or that their learning could be attenuated compared to typically developed individuals (e.g., [19]). Interestingly, it has also been proposed by Bushwick [20] that the traits characterizing ASC, and its etiology, could result from atypical social learning processes.

According to the Broader Autism Phenotype (BAP) view, it is possible to quantify the ASC traits also in the general population since the ASC is considered to be a part of a continuum of social communication difficulty (e.g., [21]). Specifically, it has been proposed that individuals with normal intelligence from the general population can lie on this continuum showing different degrees and severity of difficulties of social abilities (e.g., [22–25]). In other words, the BAP is a phenotype characterized by cognitive and behavioral characteristics qualitatively similar to ASC, but milder and below the diagnostic threshold needed to make a formal diagnosis of ASC [24, 25]. Originally, evidence of the existence of the BAP was found and observed within the family of people with ASC (e.g., [26]). Then, subsequent studies have demonstrated that autistic traits are present in the general population as well, and are continuously distributed (e.g., [23, 25]). It is possible to measure the presence of autistic traits also in typically developed individuals who have not received a formal diagnosis of ASC by administering short self-assessment questionnaires such as the “Autism Spectrum Quotient (AQ)” one [22]. It investigates five domains (i.e., social skill, attention switching, attention to detail, communication, and imagination) and returns a score indicating the presence of autistic traits, the higher the score the more the person could show autistic-like behaviours.

Relevant to the present study, Bayliss and Tipper [27] also reported a negative relationship between the magnitude of the GCE and the Autism Spectrum Quotient. Moreover, Morgan et al. [28] recently reported that autistic traits influence the extent to which mental state attributions modulate the GCE in neurotypical adults. Therefore, there is evidence that the amount of autistic traits and social abilities (e.g., mind reading), reduced in ASC, can mediate the GCE. The aim of the present study was to investigate in the general population whether or not people with higher autistic traits show reduced race attitudes and if the GCE was influenced by the race of the cueing face. To this end, we investigated in White Italian adults if the implicit race attitude towards Black and White people (measured by the Implicit Association Test – IAT, widely used to study implicit attitudes) is predicted by the level of autistic traits (measured by the AQ questionnaire). Since as previously stated, the prerequisites for social learning, in particular, interest in social stimuli (e.g., [17]), joint attention and other social abilities such as imitation (e.g., [29, 30]) are atypical or reduced in ASC, and people with high autistic traits show difficulties in processing social signals (e.g., [31, 32]), it could be that ASC is not the only condition in which stereotypical race attitudes are attenuated (e.g., [19]). That is, individuals with higher autistic traits in the general population may also show race attitudes similar to ASC individuals. Specifically, they may have attenuated implicit attitudes resulting in a less strong implicit race attitude bias. A persistent result about implicit race attitudes is that White people have negative attitudes toward Black people (showing more positive attitudes toward their own ingroup), while Black persons do not have the same amount of ingroup preference, reflecting the influence of socially shared beliefs and evaluations. To the best of our knowledge, the link between race attitudes and autistic traits in the general population has not been investigated by previous research. Specifically, we first hypothesized that individuals with higher autistic traits would have a reduced formation of race attitudes compared to individuals with medium–low autistic traits. Therefore, we expected that in White Italian adults the level of autistic traits would predict the amount of implicit race bias, with people with higher autistic traits showing a weaker ingroup bias (i.e., attenuated more positive attitudes toward Whites than toward Blacks). This in analogy with ASC people, who were reported to have a less strong negative attitude toward the outgroup (i.e., Black people in the present study) [19, 33].

Second, due to the effect of the race group membership on the GCE [15] and the supposed modulatory effect of implicit race attitudes on the GCE, we hypothesized to find a different performance depending on the level of autistic traits in a gaze cueing task (GCT) in which both White (own ingroup) and Black (own outgroup) cueing faces were presented. In other words, we expected the AQ score to moderate the interaction between Congruency and Race. In particular, we expected that White Italian adults with higher AQ score would show a similar GCE both for Black and White cueing-faces, whereas the participants with a medium–low AQ score would show a GCE only for White faces.

## Methods

The study was approved by the Committee for Research Evaluation of the Psychology Department of the University Milano-Bicocca (RM-2020–247) and conducted in accordance with the guidelines of the Declaration of Helsinki and its later amendments or comparable ethical standards.

The data of the entire study were analyzed through quantitative statistical methods by means of the softwares R (RStudio, Version 4.3.0) and Jamovi (Version 0.9.6.1). The statistical analyses were performed using the R packages lme4 and lmerTest for the Gaze Cueing task, and using the R package lm for the Implicit Association Test. The main analyses were carried out using Linear mixed-effects models (e.g., [34]).

### Participants

Informed consent was granted by all participants prior to the start of the study. All were volunteers and were unaware of the purpose of the study.

The sample size was estimated with software G*Power Version 3.1.9.4. The largest suggested sample size was of 40 (Partial η2 = 0.058, f = 0.25, alfa = 0.05, Power = 0.8), taking as reference the study by Pavan et al. [15].

Due to the COVID-19 pandemic the study was conducted online by means of the Inquisit Millisecond Web platform. It remained available from May 21, 2020 to November 13, 2020 so as to maximize the chance to collect data from a balanced number of female and male participants but it was not the case. The potential participants were reached via a University database (the Sona System platform) and through personal contacts.

One-hundred and ninety-six adults participated in the study but only data from participants who were White and Italian, did not have any psychiatric/neurological disorder, did not receive a formal diagnosis of ASC and completed the entire study were analyzed. Over all, we included one-hundred and sixty-five participants (132 females, 33 males) with an average age of 22.9 years (M = 22.9; SD = 4.76; Females: M = 22.6; SD = 5.11; Males: M = 23.9; SD = 2.87). Twenty-nine (F = 23; M = 6) of them had an AQ score >  = 23 (cut off for high autistic traits), while the remaining 136 (F = 109; M = 27) had an AQ score <  = 22 [22, 35]. Within both groups 80% of the participants were women (AQ >  = 23: F = 23 and M = 6; AQ <  = 22: F = 109 and M = 27).

### Apparatus, stimuli and procedure

The study was created offline with the Inquisit software (Version: 4.0.10.0) [36]. It lasted about 30 min in total. The participants were invited to carry out the experiment in a quiet place.

It started with the presentation of the informed consent followed by questions about age, sex, nationality, race, manual preference, and presence of neurological/psychiatric conditions so as to collect some demographic information. The entire study consisted of three main parts presented in a fixed order: in the first part the Autism Spectrum Quotient questionnaire was administered, followed by Gaze Cueing Task, and finally by the Implicit Association Test.

Importantly, to ensure that during the Gaze Cueing Task the stimuli presented online were kept of a fixed size independently of the size or resolution of the computer monitor used by the participants an additional task was introduced lasting about 2 min. This was a crucial calibration procedure because it introduced a control on the online procedure making sure that we replicated the procedure and stimulus size used in previous studies done in the laboratory [15]. More specifically, the calibration task consisted of one trial in which the instructions and a black line stimulus were presented at the center of the screen against a white background. The starting length of the line was 250 pixels. The participant’s task was to make the line 8.6 cm long. To lengthen or shorten the line, the participants had to press the “L-key” or the “K-key”, respectively. Participants were requested to measure the line with a ruler or a card 8.6-cm long (e.g., the Italian medical card or the electronic ID card), to make sure that the line was of the required length. Each time the “L-key” or the “K-key” was pressed, the line lengthened or shortened by 2 pixels respectively. In this way, at the end of the calibration procedure it was possible to know how many pixels corresponded to 8.6 cm, and to use the ratio between pixels and millimeters of each screen to scale the size of the stimuli presented, an adjustment made automatically by the software, maintaining constant the size of the stimuli seen by each participant.

### Autism-Spectrum Quotient questionnaire

The Italian version of the Autism-Spectrum Quotient questionnaire [22], translated and validated by Ruta et al. [37], was used. It is composed of 50 questions investigating autistic-like behaviours to which participants have to answer choosing between four options “Definitely agree”, “Slightly agree”, “Slightly disagree”, “Definitely disagree”. Its compilation took about 5 min. The score obtained with this questionnaire permitted us to have a quantitative measure of the level of autistic traits of our participants (i.e., AQ score).

### Gaze Cueing Task

Participants were invited to sit at about 57 cm (i.e., about 3 spans) from the computer monitor and to keep their fixation on a central cross for the whole duration of each trial, presented before each trial. They were asked to use two fingers of one hand to press the response keys.

**Fig. 1:**
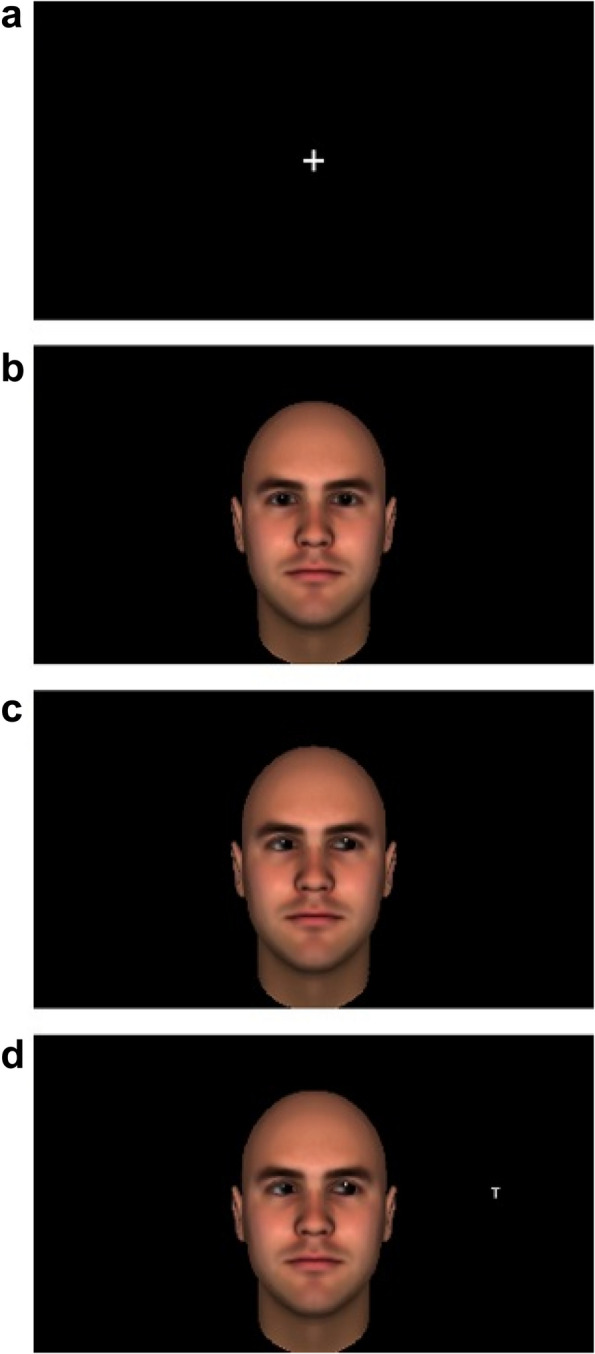
The sequence of events in an experimental trial started with a white fixation cross lasting for 900 ms (image **a**), then a face looking forward appeared remaining on the screen for others 900 ms (image **b**), when it was superimposed by the same face looking to the left or to the right (image **c**), 200 ms later a white target letter (i.e., an “L” or a “T”) appeared either on the left or on the right of the face (image **d**). In this example, the avatar of the cueing face was a White man and the gaze was averted toward the position in which the target letter appeared. Stimuli are drawn to scale. The cueing-face is taken from the database created by Pavan et al. [15]

The same stimuli employed by Pavan et al. [15] were used both in the Gaze Cueing Task and in the IAT. They consisted of avatar faces of 4 Black women, 4 Black men, 4 White women, 4 White men. More precisely, by White face we intend "Europeans with fair-skin faces" and by Black face "Africans with dark-skin faces". For each face there were 3 images, one with the eyes looking straight, one with the gaze averted to the left or to the right (Fig. 1).

The stimuli were of the same dimension as in Pavan et al. 's study [15], subtending a visual angle of 16.8° in height and 14.4° in width. They were presented against a black background. Moreover, we matched all the stimulus images for luminance, using Shine Color toolbox [38] with the software Matlab (R2019b Update 4 (9.7.0.1296695)).

**Fig. 2:**
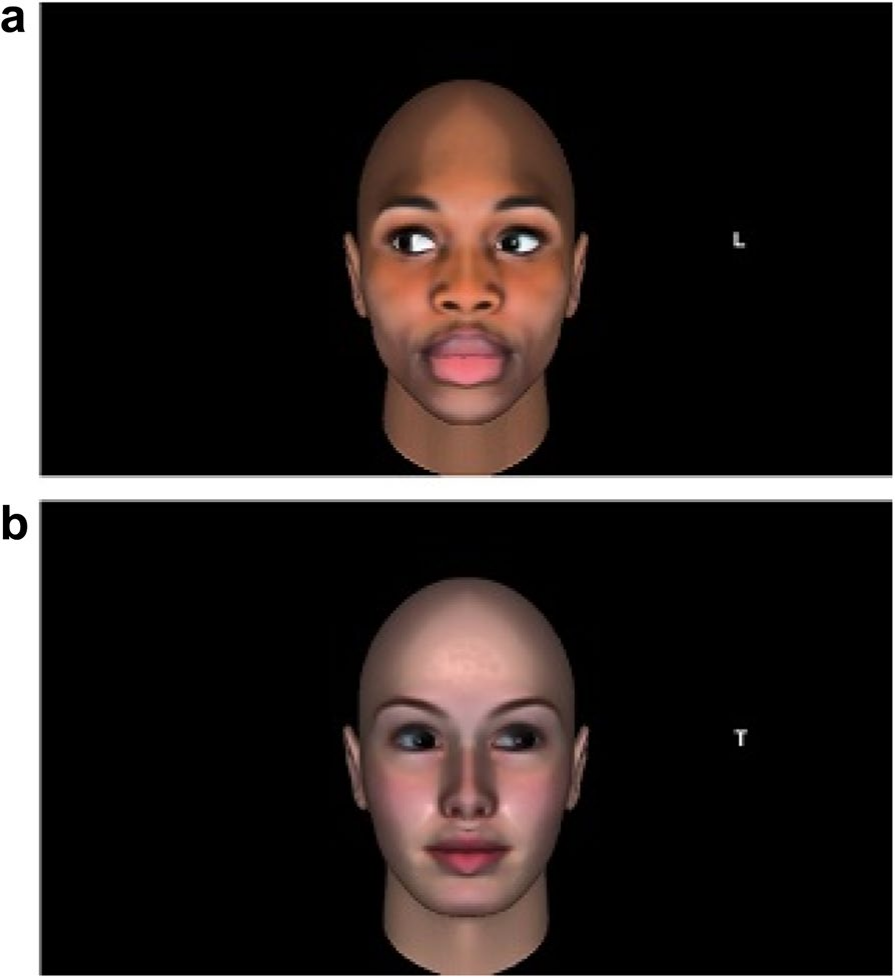
Image **a** is an example of an incongruent trial with a Black cueing face, in which the letter appeared in the opposite direction of the one indicated by gaze direction; image **b** is an example of congruent trial with a White cueing face, in which the direction of the averted gaze and position of the target letter were the same. Stimuli are drawn to scale. The cueing-faces are taken from the database created by Pavan et al. [15]

Each trial started with a white fixation cross “ + ” lasting for 900 ms, then a face looking straight ahead appeared and lasted for another 900 ms, then it was superimposed by the same face looking to the left or to the right. After 200 ms a target letter (either “L” or a “T”, written in white and with a 24-point “Arial” bold font) appeared against a black background either on the left or on the right of the face with equal probability. It appeared at 11° from the center of the screen, at the same height of the center of the screen and of the cueing face’s eyes; the target lasted (together with the face with the averted gaze) until response (see Fig. 1 for a schematic representation of the sequence of events). The target location could be 50% of the time spatially congruent or incongruent with gaze direction (see Fig. 2 for examples of incongruent and congruent trials). Participants were informed that gaze direction was not informative to where the target letter would appear and were told to ignore the face.

The participants’ task was to discriminate the target letter as quickly and accurately as possible by pressing either the “space bar” or the “J-key” on the computer keyboard with two different fingers of the same hand. Half of the participants were instructed to press the “spacebar” if the target letter was an “L” and the “J-key” when the target letter was a “T” (Mapping 1). Whereas for the other half of the participants the stimulus–response mapping was reversed (Mapping 2).

The experiment was preceded by 8 practice trials to familiarize the participants with the task (one for every possible combination of the race of the avatar, gaze averted towards the right/left, and congruency of the gaze with the location of the target letter). Only in the practice trials participants were presented with a red “X” appearing at the center of the screen when they made a mistake. Practice trials were excluded from the analysis. The experiment itself was composed of 256 trials, 64 trials for each combination of the congruency between gaze direction and target location (congruent vs. incongruent) and the race of the face (White vs. Black). The order of presentation of the trials was randomized. Participants could take a break after 128 trials. The experiment duration was about 20 min.

### Implicit Association Test

**Fig. 3:**
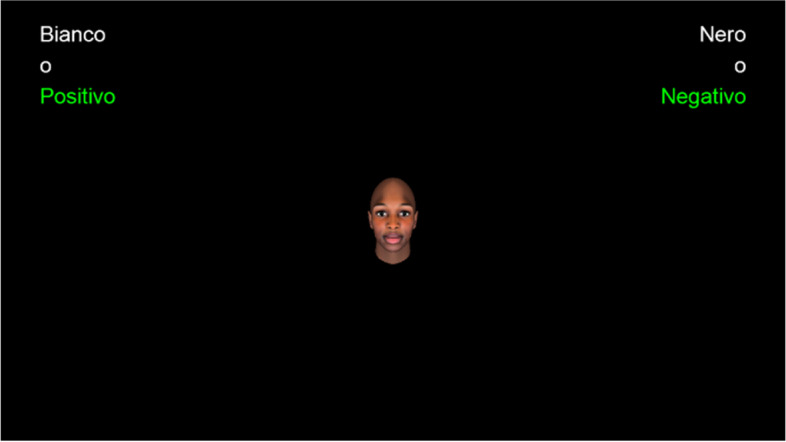
The figure illustrates an example of a compatible double-categorization trial, in which a face stimulus was shown. Participants had to press the “E-key” when a White (Bianco) face or a positive word was presented and the “I-key” when there was a Black (Nero) face or a negative word. Stimuli are drawn to scale. The stimulus (i.e., a face) is taken from the database created by Pavan et al. [15]

**Fig. 4:**
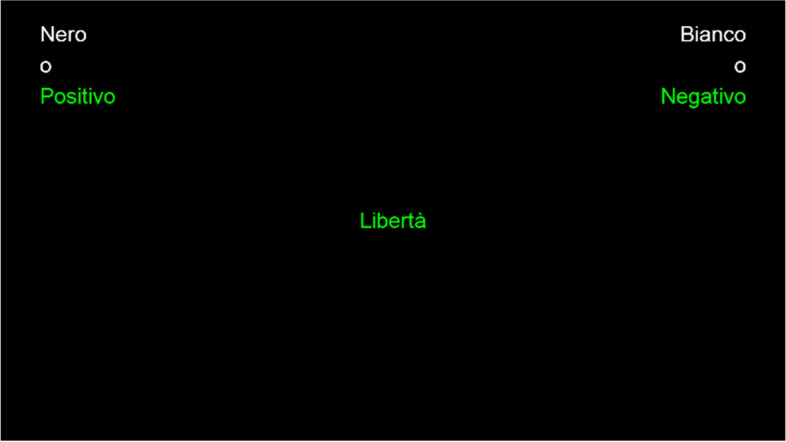
The figure represents an example of an incompatible double-categorization trial, in which a word stimulus was shown. Participants should press the “E-key” when a Black face or a positive (Positivo) word was presented and the “I-key” when there was a White face or a negative (Negativo) word. Stimuli are drawn to scale

The Implicit Association Test [39] was used to assess the participants’ implicit attitude towards the race group of the cueing faces. Participants were asked to classify the stimuli (i.e., faces and/or words) as belonging to the categories “Black” / “White” in the case of the faces, and “Positive” / “Negative” in the case of words by pressing the associated response keys on the computer keyboard (i.e., “E” or “I”) with their index fingers. The faces consisted of six faces of Black avatars and six of White ones, all with direct gaze, chosen randomly from the database created by Pavan et al. [15] and matched for luminance. The size of the stimuli was adapted to the computer monitor used by the participants so as to be the 20% of the screen both in height and in width. The word stimuli were chosen from the set used by Greenwald, McGhee and Schwartz [39] and translated into Italian, six were positive words (i.e., freedom, health, love, pleasure, rainbow, lucky^1^) and six were negative ones (i.e., death, poison, tragedy, sickness, grief, agony^2^). In each trial, the stimuli appeared one at the time. After a response was made (either correct or wrong) a new stimulus appeared after 400 ms. When the participants made a wrong response a red “X” was presented at the center of the screen for 300 ms. The test consisted of seven blocks in total. The participants first completed 40 single-categorization trials divided in two blocks. In the first block (i.e., 20 trials), the stimuli were only faces of White and Black avatars (10 each) presented in random order and the participants had to classify them as “White” or “Black” by pressing the “E-key” in the former case and the “I-key” in the latter one (Compatible target block). Instead, in the second block (i.e., 20 trials), the stimuli were only positive and negative words (10 each) appearing in random order and they were to be classified as “Positive” or “Negative” by pressing the “E-key” or the “I-key” respectively (Attribute block). Then, participants completed the third and fourth block (i.e., the Compatible blocks), which were double-categorization blocks consisting of 60 trials in total (i.e., the first 20 trials were part of the Practice Compatible block; the remaining 40 trials were part of the Critical Compatible block). In these blocks, participants had to press the “E-key” when White faces or Positive words appeared on the screen, and the “I-key” when Black faces or Negative words were presented. Trials in which the stimulus was a face and trials in which the stimulus was a word were alternated, which kind of face (i.e., either White or Black) or word (i.e., either positive or negative) was presented was chosen randomly; the same was true for the other blocks with a double categorization. Refer to Fig. 3 for an example of a compatible double-categorization trial. Next, in the fifth block (i.e., the Incompatible target block), other 40 single-categorization trials were completed in which Black and White face stimuli (20 each) were presented in random order and classified by the participants as “Black” or “White” as in the first block, but the response keys assigned to the target categories were switched (i.e., participants had to press the “E-key” for Black faces and the “I-key” for White faces). Finally, the participants completed the sixth and seventh block (i.e., the Incompatible blocks), which were double-categorization blocks consisting of 60 trials in total (i.e., the first 20 trials were part of the Practice Incompatible block; the remaining 40 trials were part of the Critical Incompatible block) in which the pairings of the categories were reversed. That is, the participants were instructed to press the “E-key” when Black faces or Positive words were presented, and the “I-key” for White faces or Negative words. Refer to Fig. 4 to see an example of an incompatible double-categorization trial. Table 1 summarizes the sequence of blocks used in the present study. The order of the Compatible and Incompatible double-categorization blocks (i.e., having first the Compatible blocks and then the Incompatible ones, or vice versa) was counterbalanced between participants. Then, based on the participants’ IAT performance, for each participant, the D score was calculated [40] by subtracting the average response latency in the Compatible blocks from the one obtained in the Incompatible blocks and dividing this difference by an inclusive standard deviation of the participant’s response latencies in the Compatible and Incompatible blocks. In the present study, a positive D score reflected the preference for White people over Black ones.

**Table 1:** Representation of the sequence of the blocks of the IAT (first column), the categorizations that participants were asked to make for each block (second column), the number of trials in each block (third column), and the response keys associated with the categories for each block (fourth column)

Blocks	Categorizations	Number of trials	Response keys
1. Compatible target	White vs. Black	20	“E-key” for White faces; “I-key” for Black faces
2. Attribute	Positive vs. Negative	20	“E-key” for positive words; “I-key” for negative words
3. Practice Compatible double-categorization	White + Positive vs. Black + Negative	20	“E-key” for White faces or positive words; “I-key” for Black faces or negative words
4. Critical Compatible double-categorization	White + Positive vs. Black + Negative	40	“E-key” for White faces or positive words “I-key” for Black faces or negative words
5. Incompatible target	Black vs. White	40	“E-key” for Black faces; “I-key” for White faces
6. Practice Incompatible double-categorization	Black + Positive vs. White + Negative	20	“E-key” for Black faces or positive words; “I-key” for White faces or negative words
7. Critical Incompatible double-categorization	Black + Positive vs. White + Negative	40	“E-key” for Black faces or positive words; “I-key” for White faces or negative words

### Data cleaning

We included in the analyses only the participants who met the criteria for being included in the analyses of both the IAT and the gaze cueing tasks so as to have the same participant sample for the analyses of the two tasks. In doing so, we aimed to make the performance at both tasks informative on the link between the presence of implicit race attitudes (i.e., ingroup preference) and its influence on the gaze cueing task. As the result, the data of 9 (F = 8; M = 1) participants^3^ were excluded from the subsequent analysis because in the Gaze Cueing Task they had an error rate (i.e., ratio of the incorrect trials on the total trials) of more than 10%, generally considered too high in a discrimination task, then leaving 156 (F = 124; M = 32) participants.

As suggested by Greenwald, Nosek and Banaji [40], we did not include in the calculation of the D score trials with reaction times (RTs) greater than 10 s (only one trial was not included in the calculation^4^) and, since none of the participants had more than 10% of the trials with RTs less than 300 ms, we included all the 156 participants in the analyses.

In the Gaze Cueing Task analysis, we did not include the trials in which reaction times for correct responses were + -2.5 Standard Deviation (SD) from the mean calculated for each participant in each condition (i.e., Congruency by Race).^5^

## Results

### Descriptive analyses of the clean sample

The mean age of the new sample was 22.9 years (*SD* = 4.89). The mean AQ score of the sample was 16.2 (*M* = 16.2; *SD* = 6.48). In the present study, we considered AQ score levels that deviate from the mean by only one standard deviation as medium–low (-1SD) and medium–high (+ 1SD) levels, while scores that deviate more strongly from the mean (+ 2SD) as high levels (see [41] for a similar procedure). It is to note that the present way to classify the levels of the AQ score matches well the classification proposed by Baron Cohen and colleagues [35].^6^

### Analyses

#### Implicit association test analyses

We calculated the D score following the guidelines by Greenwald, Nosek and Banaji [40]. The mean D score of the all sample was 0.63 (*SD* = 0.41), a positive number significantly different from zero (*M* +—*SE* = 0.63 +—0.03; *t* (155) = 18.9, *p* < 0.001), reflecting a stronger association between the concepts "White + Positive" (and "Black + Negative") than between the concepts "Black + Positive" (and "White + Negative"), therefore showing a negative attitude of our participants towards Black people.

We tested whether the implicit race attitude (i.e., D score) differed as a function of the AQ score, keeping constant the difference due to Gender, by means of a Linear Regression. The results showed that the AQ score did not significantly predict the D score (partial η^2^ = 0.004; *p* = 0.448) (R^2^ = -0.009; F (2, 153) = 0.35; *p* = 0.706).

#### Gaze Cueing task analysis

The mean error rate of the all sample was 3.6% (*M* = 3.6%; *SD* = 2.28), since the error rate was low, errors were discarded from further analyses.

We performed a Linear Mixed Model (LMM) analysis on the logarithm of the RTs^7^ for correct responses with *Congruency* (congruent vs. incongruent), *Race* (White vs. Black gaze cueing faces), *AQ score*, and the interactions between the three as fixed effects. We also inserted *Gender* (female vs. male) in the model, without interactions, to calculate the effects of the other independent variables while covariating Gender. A random intercept for subject was included to account for within-subject correlations.

**Fig. 5:**
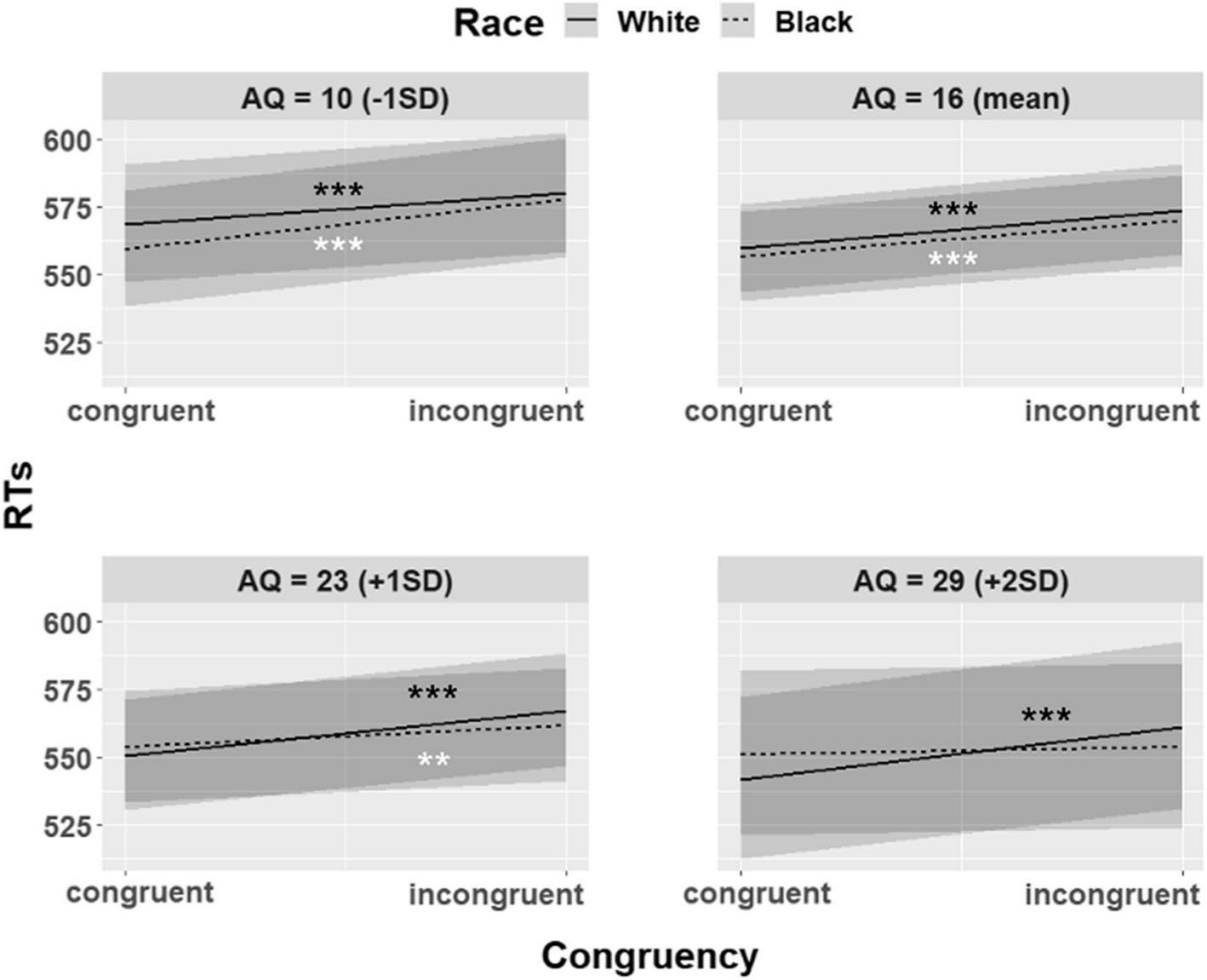
The graph shows the interaction between the Congruency, Race, and AQ score (*F* (1, 37,302) = 10.73; *p* = .001). Specifically, it shows how the interaction between Congruency and Race changes at the various levels of the AQ score, that are: AQ = 10 (-1SD) in the upper left panel; AQ = 16 (mean) in the upper right panel; AQ = 23 (+ 1SD) in the lower left panel; AQ = 29 (+ 2SD) in the lower right panel. The graph shows that at medium–low, medium, and medium–high AQ score levels (i.e., AQ = 10; AQ = 16; AQ = 23) participants were faster in the congruent condition than in the incongruent one, with both White (AQ = 10: *ratio* = 0.98; *p*_Bonferroni_ < .0001; AQ = 16: *ratio* = 0.98; *p*_Bonferroni_ < .0001; AQ = 23: *ratio* = 0.97; *p*_Bonferroni_ < .0001) and Black gaze-cueing faces (AQ = 10: *ratio* = 0.97; *p*_Bonferroni_ < .0001; AQ = 16: *ratio* = 0.98; *p*_Bonferroni_ < .0001; AQ = 23: *ratio* = 0.99; *p*_Bonferroni_ = .009). Whereas at high AQ score level (AQ = 29, lower right panel) participants were faster in the congruent condition than in the incongruent one only with White faces (*ratio* = 0.97; *p*_Bonferroni_ < .0001; *M* = 542; *SE* = 15.2; 95% CI [513, 572] vs. *M* = 561; *SE* = 15.7; 95% CI [531, 593]) and not with Black ones (*ratio* = 0.995; *p*_Bonferroni_ = 1; *M* = 551; *SE* = 15.4; 95% CI [522, 582] vs. *M* = 554; *SE* = 15.5; 95% CI [524, 585]). The shaded areas around lines represent the 95% confidence intervals. Black asterisks are referred to the solid lines, white asterisks to the dashed lines. ** *p* < .01, *** *p* < .001

The LMM showed a significant main effect of Congruency (*F* (1, 37,302) = 119.13;* p* < 0.001; partial η^2^ = 0.003), due to faster RTs for congruent (*M* = 558; *SE* = 8.24; 95% CI [542, 574]) than incongruent condition (*M* = 572; *SE* = 8.44; 95% CI [555, 588]). The main effect of Race was also significant (*F* (1, 37,302) = 7.73; *p* = 0.005; partial η^2^ = 0.0002), participants were weakly faster with Black gaze cueing faces (*M* = 563; *SE* = 8.31; 95% CI [547, 580]) than with White faces (*M* = 566; *SE* = 8.37; 95% CI [550, 583]). More importantly, the three-way interaction Congruency x Race x AQ score was also significant (*F* (1, 37,302) = 10.73; *p* = 0.001; partial η^2^ = 0.0003). In particular, the Post-Hoc analysis with Bonferroni correction showed that at medium–low (i.e., AQ = 10^8^), medium (AQ = 16^9^), and medium–high levels of the AQ score (AQ = 23^10^), participants had a significant GCE for both White (AQ = 10: *ratio*^11^ = 0.98; *p*_Bonferroni_ < 0.0001; 95% CI [0.97, 0.99]; congruent vs. incongruent) (AQ = 16: *ratio* = 0.98; *p*_Bonferroni_ < 0.0001; 95% CI [0.97, 0.98]) (AQ = 23: *ratio* = 0.97; *p*_Bonferroni_ < 0.0001; 95% CI [0.96, 0.98]) and Black faces (AQ = 10: *ratio* = 0.97; *p*_Bonferroni_ < 0.0001; 95% CI [0.96, 0.98]; congruent vs. incongruent) (AQ = 16: *ratio* = 0.98; *p*_Bonferroni_ < 0.0001; 95% CI [0.97, 0.99]) (AQ = 23: *ratio* = 0.99; *p*_Bonferroni_ = 0.009; 95% CI [0.98, 1]). Specifically:

- At AQ = 10 level:
  Participants were faster in the congruent trials than in the incongruent ones with both White (*M* = 569; *SE* = 11; 95% CI [547, 591] vs. *M* = 580; *SE* = 11.2; 95% CI [558, 602]) and Black faces (*M* = 559; *SE* = 10.8; 95% CI [538, 581] vs. *M* = 578; *SE* = 11.2; 95% CI [556, 600]).
- At AQ = 16 level:
  Participants were faster in the congruent trials than in the incongruent ones with both White (*M* = 559; *SE* = 8.31; 95% CI [543, 576] vs. *M* = 574; *SE* = 8.52; 95% CI [557, 591]) and Black faces (*M* = 556; *SE* = 8.26; 95% CI [540, 573] vs. *M* = 570; *SE* = 8.46; 95% CI [553, 586]).
  Participants were faster in the congruent trials than in the incongruent ones with both White (*M* = 550; *SE* = 10.4; 95% CI [530, 571] vs. *M* = 567; *SE* = 10.7; 95% CI [547, 589]) and Black faces (*M* = 554; *SE* = 10.5; 95% CI [534, 575] vs. *M* = 562; *SE* = 10.6; 95% CI [541, 583]).
- At AQ = 23 level:
  Whereas at high levels of the AQ score (AQ = 29^12^), participants had a significant GCE only for White faces (*ratio* = 0.97; *p*_Bonferroni_ < 0.0001; 95% CI [0.95, 0.98]; *M* = 542; *SE* = 15.2; 95% CI [513, 572] vs. *M* = 561; *SE* = 15.7; 95% CI [531, 593]) but not for Black ones (*ratio* = 0.995; *p*_Bonferroni_ = 1; 95% CI [0.98, 1.01]; *M* = 551; *SE* = 15.4; 95% CI [522, 582] vs. *M* = 554; *SE* = 15.5; 95% CI [524, 585]). Figure 5 shows how the interaction between Congruency and Race varies at the various levels of the AQ score.

#### LMM with Gender’s interactions

To understand if Gender might influence the three-way interaction of interest (i.e., *Congruency* x *Race* x *AQ score*), the same analysis (LMM) was conducted on the logarithm of the RTs for correct responses with the same factors as before but adding *Gender’*s interactions in the model, focusing on the four-way interaction (i.e., *Congruency* x *Race* x *AQ score* x *Gender*) and the three-way interaction of interest. A random intercept for subject was included to account for within-subject correlations.

The four-way interaction between *Congruency*, *Race*, *AQ score*, and *Gender* was not significant (*F* (1, 37,296) = 0.83; *p* = 0.363; partial η^2^ = 0.00002), whereas the three-way interaction *Congruency* x *Race* x *AQ score* was still significant (*F* (1, 37,296) = 4.33; *p* = 0.038; partial η^2^ = 0.0001). The interaction between Congruency, Race, and AQ score did not change at different levels of the variable Gender and it remained significant when Gender’s interactions were added to the model.

## Discussion

The aim of our study was two-fold. On the one hand, with the use of the Implicit Association Test [39], we investigated whether racial attitude formation is reduced in individuals with higher autistic traits; on the other hand, with the use of a Gaze Cueing task [3], we studied whether autistic traits and race bias can modulate the gaze cueing effect.

The main result concerning the Implicit Association Test is that participants showed a more positive implicit attitude toward White people (i.e., their ingroup) than toward Black people (i.e., their outgroup). The size of the race bias was not predicted by the level of the AQ score. This finding contradicts our first hypothesis according to which a reduced ingroup bias should be present in participants with higher autistic traits. In fact, the amount of ingroup bias did not change depending on the quantity of autistic traits. The all sample had a preference for their ingroup, showing a more positive attitude towards White people than Black ones. It seems that the reduced formation of implicit race attitudes, previously found in the ASC population consisting of an attenuated race bias [19, 33], does not extend to the general population with high autistic traits. The less effective social learning of attitudes that has been identified as a possible cause of a reduced race bias formation, and has served as the starting point in studies on the attitudes of the ASC population (e.g., [19]), does not seem to be present, or have an effect in our sample. In the present study, in which autistic traits have been taken into account, we found a race bias that is in line with past researches done on White people and White Italians, in particular, in the general population [13, 14, 42]. It should also be said that cultural differences and other factors such, for instance, the level of education or socioeconomic status, which were not taken into account in the present study, may have a greater influence than the level of autistic traits on race bias formation. Moreover, it is noteworthy that our sample was taken from the general population and none of the participants had a formal diagnosis of ASC. Future research should extend the present study to individuals with ASC and systematically assess social learning.

The second aim of the study was to investigate the effect of autistic traits and implicit ingroup bias on the GCE. In the Gaze Cueing task, the GCE emerged, with participants being faster in the congruent trials (i.e., the gaze averted towards the location in which the target appeared) than in the incongruent ones.

Interestingly, the analysis showed that the significant GCE was modulated by the AQ score. Specifically, the interaction between Congruency and Race was modulated by the AQ score. Participants with high AQ score showed a significant GCE only with White faces, while participants with medium–low, medium, and medium–high AQ score showed it with both White and Black gaze cueing faces.

Contrary to what one could expect from the results found in the IAT, showing the presence of an implicit race bias, the race of the cueing face per se, and the gender of the participants, did not modulate the GCE. This is in contrast with the results reported by Pavan et al. [15] who found in White Italian adult participants a GCE only with White cueing faces. However, Pavan et al. [15] did not systematically measure either the AQ score or the implicit race attitude and the gender of the participants was not taken into account.^13^ Methodological differences, thus, may account for the discrepancy of our and their results.

The significant interaction between Congruency, Race, and AQ score, that emerged in the present study showing that participants with high AQ score exhibited a significant GCE only for White faces, is opposite to what we initially expected, first because we thought that an attenuated race bias was present in the high autistic traits participants and then it would result in a GCE for both White and Black faces. One could reason, in fact, that individuals with high levels of autistic traits behave differently in joint attention task than those with medium and low level of autistic traits since the presence of more autistic traits is supposed to result in attenuated joint attention skills. As stated in the introduction, people with high autistic traits have some difficulties in processing social signals (e.g., [31, 32]), and these difficulties can extend to having attenuated attention toward social cues (e.g., [43]) and consequently a reduced joint attention behaviour. Interestingly, however, this seems to be the case only with the Black faces who, in the present study, also represent the outgroup. A possibility explaining the present result could be that high autistic traits individuals retain the ability to orient to gaze direction. Nevertheless, they may have a more limited capacity of social attention and, therefore for this reason, they restrict their attentional orienting by prioritizing only one type of social stimulus. That is, in the present study, only the ingroup faces guide orienting of attention (i.e., White faces) likely because they are more functional for successful social interactions since they elicited more positive attitudes and are more familiar. Indeed, it has been shown that faces more similar or more familiar to participants elicit a larger GCE [8, 44]. In future studies, it would be important to test these alternative explanations, using multiracial faces other than Black ones, and measuring familiarity more systematically.

There are two main shortcomings in our study. First of all, the imbalance in terms of the numerosity between female and male participants. However, many studies in the GCE literature present a gender imbalance, which should be acknowledged (e.g., [27, 45, 46]) and tested a much smaller sample than the one reported in the present paper [47].

Another limitation is that the present study has been conducted online during the COVID-19 pandemic, this meant having less control on variables other than the ones investigated, which could have affected the findings and may have introduced some biases, explaining also the lack of replication of previous studies (e.g., [15]). In a similar vein, our participants did not carry out the study all in the same controlled setting (laboratory), but each one carried it out in a different environment (e.g., their apartment) and a different context may have played some role in the results. However, there is evidence of the validity and reliability of conducting online experiments recording not just accuracy but also RTs (e.g., [48, 49]). Nevertheless, it is worth replicating the study in presence in the laboratory balancing the gender of participants.

We believe that the influence of the race attitudes on the gaze-cueing effect deserves to be studied further to clarify the existence of a modulatory effect of the shared racial group membership on the GCE. And, if confirmed, in which direction it goes. The present findings can be considered as preliminary and suggest that individuals who reported an implicit preference towards their ingroup at the IAT also show a GCE for the same cueing faces (i.e., White faces). However, it is not always true that they do not orient their attention in the direction of gaze of the individuals toward which they show an implicit negative bias (i.e., Black people). Instead, GCE seems to be modulated by the level of autistic traits. To the best of our knowledge, the present study is the first one investigating joint attention by bringing together the effect of autistic traits and the measurement of the implicit race attitude, two important aspects for social interactions.

In conclusion, our findings suggest that White Italian participants have an implicit negative attitude toward Black people, that isn’t influenced by the level of autistic traits. Intriguingly, this negative attitude seems to affect orienting of attention only of people with high autistic traits, who shift their attention in the direction of the gaze of White cueing faces but not of Black ones. The ingroup bias, thus, seems to influence the gaze-cueing effect of a population previously shown to have reduced attention to social cues (e.g., [43]) by prioritizing joint attention with ingroup members.

## Data Availability

The datasets generated for this study are deposited on University of Milano—Bicocca data repository (Bicocca Open Archive Research Data, 10.17632/rj726bg5pk.1). All the data have been de-identified to preserve the privacy of research participants. The corresponding author will grant the permission to access all the files on request. No ad-hoc questionnaire, survey and/or interview guide have been developed for the present study.

## Abbreviations

ANOVA: Analysis of variance
AQ: Autism Spectrum Quotient
ASC: Autism Spectrum Condition
BAP: Broader Autism Phenotype
CI: Confidence interval
COVID-19: Coronavirus disease of 2019
GCE: Gaze-cueing effect
GCT: Gaze cueing task
IAT: Implicit Association Test
ID: Identification card
LMM: Linear Mixed Model
M: Mean
RTs: Reaction Times
SD: Standard Deviation
SE: Standard Error
USA: United States of America

## Footnotes

1 The positive words translated: Libertà, salute, amore, piacere, arcobaleno, fortunato.

2 The negative words translated: Morte, veleno, tragedia, malattia, dolore, agonia.

3 The AQ scores of the excluded participants were, in ascending order: 11; 12; 14; 18; 20; 21; 23; 23; 26.

4 0.005% of the trials were excluded from the calculation of the D score.

5 2.7% of the trials were excluded (i.e., 1034 trials of a total of 38,498).

6 AQ score classification: Low = 0–10 AQ score; Medium = 11–22 AQ score; Above average = 23–31 AQ score; Very high = 32–50 AQ score; Maximum = 50 AQ score.

7 To deal with skewed data (skewness = 2.8) we performed a log-transformation on the dependent variable RTs.

8 AQ = -1 SD.

9 AQ = mean AQ score (16.2).

10 AQ =  + 1 SD.

11 Ratio corresponds to ratio of means when back-transformation is applied.

12 AQ =  + 2 SD.

13 In order to match our experimental design with that used in Pavan et al.’s study (15) we ran a Repeated Measures ANOVA on the median RTs for correct responses with Congruency and Race as within-subject factors. This analysis showed a significant main effect of both Congruency (F (1, 155) = 79.24; *p* < .001; partial η^2^ = .34) and Race (F (1, 155) = 6.45; p = .012; partial η^2^ = .04), but no significant interaction between the two was found (F (1, 155) = 6.62e-4; *p* = .98; partial η^2^ = 0).
